# Deep Brain Stimulation for the Management of Refractory Neurological Disorders: A Comprehensive Review

**DOI:** 10.3390/medicina59111991

**Published:** 2023-11-13

**Authors:** Jamir Pitton Rissardo, Nilofar Murtaza Vora, Irra Tariq, Amna Mujtaba, Ana Letícia Fornari Caprara

**Affiliations:** 1 Neurology Department, Cooper University Hospital, Camden, NJ 08103, USA; 2 Medicine Department, Terna Speciality Hospital and Research Centre, Navi Mumbai 400706, India; nilofar031202@gmail.com; 3 Medicine Department, United Medical & Dental College, Karachi 75600, Pakistan; irratariq@gmail.com; 4 Medicine Department, Karachi Medical & Dental College, Karachi 74700, Pakistan; mujtabaamna@gmail.com; 5 Medicine Department, Federal University of Santa Maria, Santa Maria 97105-900, Brazil; ana.leticia.fornari@gmail.com

**Keywords:** DBS, movement disorder, brain stimulation, electrical stimulation, IPG, Parkinson’s disease, tremor, symptomatic, neurosurgery, neurology

## Abstract

In recent decades, deep brain stimulation (DBS) has been extensively studied due to its reversibility and significantly fewer side effects. DBS is mainly a symptomatic therapy, but the stimulation of subcortical areas by DBS is believed to affect the cytoarchitecture of the brain, leading to adaptability and neurogenesis. The neurological disorders most commonly studied with DBS were Parkinson’s disease, essential tremor, obsessive-compulsive disorder, and major depressive disorder. The most precise approach to evaluating the location of the leads still relies on the stimulus-induced side effects reported by the patients. Moreover, the adequate voltage and DBS current field could correlate with the patient’s symptoms. Implantable pulse generators are the main parts of the DBS, and their main characteristics, such as rechargeable capability, magnetic resonance imaging (MRI) safety, and device size, should always be discussed with patients. The safety of MRI will depend on several parameters: the part of the body where the device is implanted, the part of the body scanned, and the MRI-tesla magnetic field. It is worth mentioning that drug-resistant individuals may have different pathophysiological explanations for their resistance to medications, which could affect the efficacy of DBS therapy. Therefore, this could explain the significant difference in the outcomes of studies with DBS in individuals with drug-resistant neurological conditions.

## 1. Introduction

Deep brain stimulation (DBS), according to the National Institute of Neurological Disorders and Stroke (NINDS), is a surgical method used to manage various neurological conditions that do not effectively respond to conventional therapy. It comprises a neurostimulator surgically implanted battery-powered gadget, which resembles a cardiac pacemaker, that provides electrical stimulation to the appropriate location to block aberrant nerve signals [1]. The first studies with electrical stimulation of the cortex were designed at the end of the 19th century. Still, the main devices were only developed in the mid-20th century, following the scientific and technological achievements of the information age (Table 1).

The first condition approved to be managed with DBS was essential tremor in 1997 by the US Food and Drug Administration (FDA). In the following years, clinical trials were published showing the efficacy of DBS therapy for managing other movement disorders. In this context, DBS for Parkinson’s disease (PD) was approved by the FDA in 2002, and dystonia in 2003 [2]. The current guidelines recommend DBS rather than ablative surgical methods for treating drug-resistant PD. Additionally, DBS has shown promise in managing other neuropsychiatric conditions, such as substance-related and addictive disorders, aggressive behavior, eating disorders, major depressive disorder, obsessive-compulsive disorder, and refractory Gilles de la Tourette syndrome [3].

DBS implantation was based on lesioning operations performed in the last century to improve neurological symptoms, which resulted in a high percentage of undesired side effects [4]. DBS was considered a safer alternative when compared to lesioning procedures due to fewer adverse events, leading to active research and further investigation of neuromodulation approaches for various neurological disorders [5].

**Figure 1:**
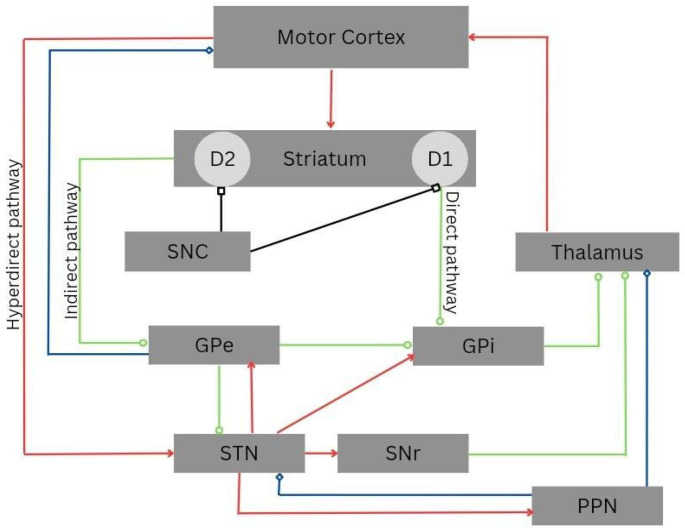
Cortico-basal-ganglia-thalamo-cortical circuitry. The direct, indirect, and hyperdirect pathways are indicated. Green lines denote inhibitory connections (GABAergic), red lines denote excitatory connections (glutamatergic), black lines denote dopaminergic pathways, and blue lines denote mixed cholinergic connections. Notably, the pedunculopontine nucleus (PPN) exhibits anatomic projections to the striatum and cortex. Abbreviations: D1, dopamine receptor D1; D2, dopamine receptor D2; GPe, external globus pallidus; GPi, internal globus pallidus; PPN, pedunculopontine nucleus; SNC, substantia nigra pars compacta; SNr, substantia nigra pars reticulata; STN, subthalamic nucleus.

Although DBS has been extensively used in managing tremor in individuals with PD, the exact mechanism of action for improving the symptoms in the neural circuitry is not fully understood. It is believed that stimulating the main nerve tracts while inhibiting the nearby neurons may facilitate the movement (Figure 1). The zones of uncertainty and cerebellar-thalamic pathways, which decrease tremor and increase dopamine, are also implicated [6]. Moreover, the nosological entity with adequate stimulation parameters and the cytoarchitecture of the brain structure (typically subcortical) are probably related to the efficacy of DBS therapy [7]. In this way, some authors proposed that the leads of DBS can inhibit the structures rich in cell bodies or disinhibit a specific collection of axons, leading to the synchronization of an abnormal pattern, which can facilitate movement or inhibit unusual neuronal activity [8]. Some individuals show a progressive improvement in motor symptoms, suggesting a possible change in the cytoarchitecture of the central nervous system and neuroplasticity [9].

## 2. Methodology

We searched six databases to locate existing reports on Deep Brain Stimulation for the Management of Refractory Neurological Disorders published until October 2023 in electronic form. Excerpta Medica (Embase), Google Scholar, Latin American and Caribbean Health Sciences Literature (Lilacs), Medline, Scientific Electronic Library Online (Scielo), and Science Direct were searched. Search terms were “movement disorder, neuropsychiatry, Parkinson’s disease, essential tremor, dystonia, obsessive-compulsive disorder, epilepsy, Tourette syndrome, major depressive disorder, eating disorders and obesity, substance abuse/addiction, chronic pain, Alzheimer’s disease, posttraumatic stress disorder, anxiety disorder, tinnitus, refractory aggression, bipolar disorder, headache, chorea, and restless legs syndrome." These terms were combined with “deep brain stimulation, DBS.”

## 3. Surgical Techniques

The surgical procedure for implanting DBS devices involves several key elements and can vary in approach and technique. A meticulous preoperative airway assessment is necessary since the patient’s head will be immobilized in a stereotactic headframe during the DBS procedure. Monitored anesthesia care with sedation is the most commonly used anesthesia technique during lead implantation for most patients. This leads to minimal effects of anesthetic agents on neuronal background and spike activity during microelectrode recording localization [10].

The primary components of a fully implanted DBS system include the precise implantation of an intracranial electrode, which involves surgically placing an intracranial electrode into the specific target area within the brain where stimulation is intended. The implant lead extension connects the intracranial electrode to the power-generating and programming sources. An internal pulse generator that generates electrical pulses for stimulation is implanted under the skin, typically in the chest or abdomen [11].

The surgical procedure for DBS can differ among medical facilities and centers. The most common approach for implanting the device is general anesthesia. On the other hand, local anesthetics are applied for device maintenance that does not involve lead manipulations such as battery changes [12]. A Leksell stereotactic frame is sometimes securely attached to the patient’s head under local anesthesia. This frame is used for precise targeting during the surgery. After securing the frame, stereotactic imaging is performed to aid in planning the electrode’s target and trajectory. Various software packages are available for this purpose, and they can employ coordinate frame-based, frameless, or robotic stereotaxic procedures [13].

Overall, the choice of approach and surgical technique may depend on the specific patient, the target area within the brain, and the preferences and expertise of the medical facility performing the procedure.

The surgical procedure for DBS involves the following steps: The patient is positioned semi-recumbent, and the scalp is prepared by clipping the hair and applying betadine solution to ensure sterility. Then, a coronally oriented incision is typically made, spanning Kocher’s point bilaterally on the scalp. However, alternative incision techniques can be used. The scalp is opened to expose the skull’s frontal bone. A hole, or trephination, is made approximately 1 cm anterior to the coronal suture and at least 2 cm lateral from the midline of the skull. The dura mater is coagulated and carefully incised. Special care is taken to minimize cerebrospinal fluid loss. A guide cannula is inserted into the brain, typically about 1.0 to 1.5 cm above the intended target area. Microelectrode recording is used to identify the electrophysiological characteristics of the target structure and determine its dorsal-ventral boundaries. This helps with the precise placement of the macrolectrodes. Once a suitable tract is identified, the microelectrodes are removed, and a permanent macroelectrode is inserted into the target structure [14]. 

Stimulation tests are conducted at each electrode contact point to evaluate for adverse effects and clinical efficacy. This ensures that the stimulation is effective and safe. The proper placement of the DBS electrode is verified through intra-operative fluoroscopy [15]. If placement is confirmed, the electrode is affixed to the skull. The incision is closed, completing the surgical procedure. These steps ensure the precise placement of the DBS electrode in the intended target area within the brain while minimizing complications. It is a delicate and highly specialized procedure performed by neurosurgeons with expertise in DBS. In some DBS centers, microelectrodes are advanced through the cannula for recording or stimulation. The microelectrode stimulation can define the anatomical location of the electrode, which can be further assessed with directional leads and changing the voltage and current. This is important for the evaluation of possible side effects related to the localization of the leads, such as paresthesia, muscle contractions, and flashes of light [16]. Also, during the insertion of the DBS lead, fluoroscopy can be used to confirm the location of the lead in a two-dimensional view (Table 2).

**Figure 2:**
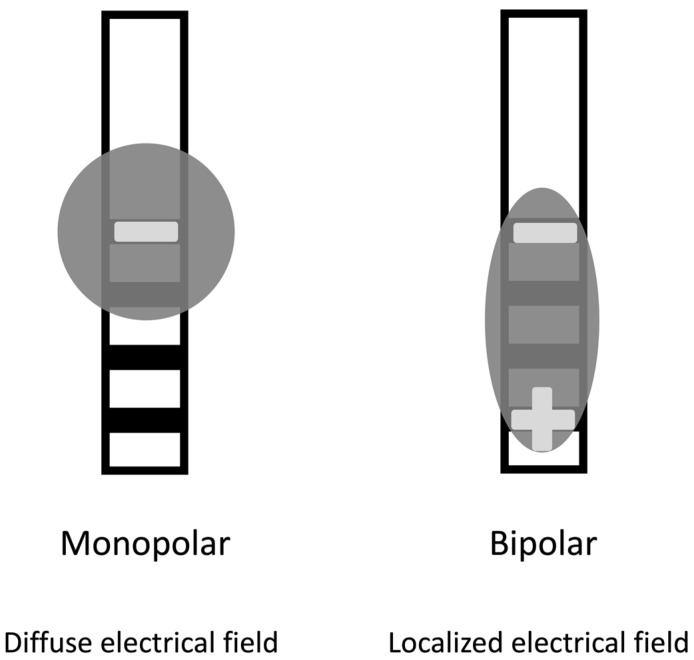
Modes of stimulation. The monopolar (cathodic) stimulation has a spreading negative current in all directions. In the bipolar, the electrode has both anodic and cathodic contact points, with a narrower and more intense flow of current between them.

Following the initial procedure, the patient undergoes general anesthesia, and the intracranial electrodes are connected to extension wires. These wires are placed under the skin, behind the ear, and down to the chest through a tunnel. An additional incision is made below the clavicle to create a pocket for the internal pulse generator. Then, the extension wires are connected to the internal pulse generator, and the system’s impedances are checked to ensure proper function [27]. Patients are usually admitted for observation for one night after the procedure. After, an outpatient appointment is scheduled within eight weeks of the procedure for device activation and programming, ensuring the DBS system is optimized for their specific needs. When treating Parkinson’s disease, programmers often begin with a monopolar configuration to stimulate the brain. In this configuration, a contact electrode is the cathode with a negative voltage. In contrast, the outer casing of the implantable pulse generator serves as the anode with a positive voltage. If adverse effects arise at higher voltages, a bipolar stimulation configuration can be used. In this configuration, one contact serves as the cathode and another as the anode, limiting current spread into adjacent brain regions that cause side effects. This technique is useful in ensuring that therapy remains within the therapeutic range and does not induce any side effects (Figure 2) [28].

The implantable pulse generators contain a battery, power module, central processing unit, program memory, and a microprocessor. They are the DBS system’s active components and control the devices’ functions, including activation, deactivation, pulsing parameters, internal diagnostics, and communication with external devices. Features of implantable pulse generators for deep brain stimulation are described in Table 3 (Table 3) [29].

## 4. Indications for DBS

According to the Core Assessment Program for Surgical Interventional Therapies in PD (CAPSIT-PD), patients being considered for surgical intervention should meet certain requirements. For atypical types of parkinsonism to meet these requirements, the disease duration should be at least five years, which often corresponds to when most patients will need levodopa therapy. Patients undergoing DBS implantation should have a good response to dopamine therapy, commonly measured as an improvement of more than 30% on the motor subscore of the Unified PD Rating Scale (UPDRS part III). For patients with severe depression and prior dementia, the CAPSIT-PD guidelines also advise against surgery [10].

It is important to stress that no evidence currently supports the use of DBS for diseases such as corticobasal degeneration, multiple system atrophy, progressive supranuclear palsy, and Lewy body dementia that can have similar clinical symptoms to PD. Therefore, before having DBS surgery, an expert in movement disorders must establish a PD diagnosis [30]. Patients with a confirmed PD diagnosis should perform a routine levodopa challenge test to evaluate their receptivity to levodopa medication and estimate the anticipated benefit following surgery, in which the percentage of motor symptoms improvement is directly associated with the clinical benefits of DBS [31]. Additionally, under the supervision of a movement disorders expert, patients should experience incapacitating dyskinesias and motor fluctuations despite using all appropriate drugs at therapeutic levels [32].

A brain MRI before surgery to rule out any structural disease in all patients requiring DBS should be conducted. A preoperative evaluation by a neuropsychiatrist and neuropsychologist is also recommended to stratify risks and identify patients who require closer postoperative follow-up, particularly those with moderate cognitive impairment or mood/behavioral abnormalities that need care [33].

Although there are no set age restrictions for DBS surgery, most patients in the early clinical trials were in the 50–65 age range, with only a few studies involving patients beyond 75. According to a recent study, surgery can still benefit elderly people without posing an additional risk [34]. However, further research is required to evaluate the long-term effects of DBS surgery in PD patients over sixty [35]. Some assumptions exist about mild positive outcomes in this subgroup of individuals undergoing DBS therapy due to other comorbidities’ influence on the patient’s functional spectrum [34].

Studies assessing the DBS complication rates failed to identify any correlation between advancing age and the percentage of adverse events within 90 days of the procedure [36]. More research is required to predict the long-term effects of DBS surgery on the elderly population with PD. Recent studies showed that DBS may be helpful even before motor symptoms become severe [37]. This contradicts the idea that surgery should only be considered in severe instances and emphasizes the significance of a precise diagnosis. Before surgery, the patient’s medical conditions and medication list should be carefully assessed. Particular attention should be paid to coronary vascular disease, anticoagulant use, hypertension, diabetes, age, and general biophysical profile. Since DBS is an additional therapy intended to improve motor symptoms rather than cure them, providing counseling that meets patient expectations is critical [38].

A multidisciplinary team with experience in DBS, comprising a neurologist with competence in movement disorders, a competent neuropsychologist, and a neurosurgeon trained in stereotactic and functional neurosurgery, is crucial for optimal results. It is worth remembering that DBS procedures are more likely to have favorable postoperative outcomes in facilities that execute a moderate-to-high volume of surgery for movement disorders [39].

The results of the clinical trials related to DBS are registered on ClinicalTrials.gov (Table 4). PD is the neurological condition with the largest number of clinical trials with DBS, in which more than three hundred studies were identified.

### 4.1. Parkinson’s Disease

The most frequent indication for DBS therapy is PD. Its primary objective is to alleviate the motor symptoms of PD. However, its effectiveness in addressing non-motor symptoms such as cognitive and other neuropsychiatric conditions in individuals with PD should still be investigated [40]. The first studies performed with DBS in PD reported no improvement in non-motor symptoms. Still, more recent studies have revealed a significant improvement in the long-term follow-up of these individuals. This can be partially explained by the neuroplasticity stimulated by the DBS device.

DBSs most common anatomical targets for managing tremors in individuals with PD are the globus pallidus pars interna (GPi) and the subthalamic nucleus (STN). The improvement of the motor subscores of the UPDRS greatly varies from 25 to 60%. Also, the changes in the levodopa equivalent doses were approximately 50% with the DBS of the STN [41]. Interestingly, no statistically significant differences between GPi and STN targets were found in the management of tremors. However, it is important to note that STN DBS may have more significant drawbacks due to its ventral position compared to GPi, including visual disturbances and mood changes [42].

Apparently, DBS of the ventral intermediate thalamic nucleus (Vim) is the most effective target for individuals presenting with tremor-predominant PD [43]. Currently, DBS techniques are not indicated for gait and cognitive disorders, but there is ongoing exploration of alternative brain regions to improve these aspects. For instance, the pedunculopontine nucleus (PPN) has been investigated as a target to manage gait disorders [44], and the nucleus basalis of Meynert (nbM) is under examination as a potential target for addressing cognitive impairment [45]. It is worth mentioning that these two clinical manifestations may be secondarily affected by the key features of PD, which may show improvement after the amelioration of the basilar symptoms of PD.

The differentiation between PD and other forms of parkinsonism is important for the surgical management of the pathologies. Apparently, only PD will benefit from DBS. Brusa et al. showed an anecdotal benefit of DBS of the pedunculopontine nucleus in a subject diagnosed with progressive supranuclear palsy with predominant parkinsonism [46]. However, in a clinical trial with eight individuals with long-term follow-up, no benefit of DBS in the motor symptoms of progressive supranuclear palsy with Richardson syndrome was observed [47].

The main indications for DBS are motor fluctuations and dyskinesias in patients with PD. Also, although Vim is commonly recommended for tremors, the most disabling features of PD are dyskinesias and motor fluctuations. Therefore, most procedures will target GPi and STN [41]. The mechanism related to improving symptoms with DBS is not fully understood (Table 5). It is believed that the DBS modulation in the excitation of STN leads to reduced thalamocortical drive, which facilitates the movement. Moreover, higher-frequency DBS was observed to decrease neuronal activity and increase efferent fiber pathways [46].

### 4.2. Essential Tremor

Essential tremor is believed to affect more than five percent of the population. It is characterized by isolated head tremor or bilateral postural tremor of the hands, which can be kinetic or postural. The first studies with DBS in essential tremor were performed in the early 1980s, but only two decades later, DBS for managing essential tremor was approved by the FDA [48]. Some centers only recommend surgical intervention after the failure of first-line and second-line therapies [49].

The most common and first target studied for managing essential tremor was the Vim nucleus [50]. Other targets, such as the posterior subthalamic area and the caudal zona incerta, have also been investigated to manage the essential tremor motor symptoms. However, the data available for these targets are limited [51]. 

The reduction of kinetic tremor in essential tremor with DBS of the Vim nucleus ranges from 53% to 63%. Bilateral Vim DBS, also considered safe, achieves an even more substantial overall tremor reduction of 66–78%. Interestingly, these significant benefits of DBS of the bilateral vim nuclei were also observed with axial, head, and voice tremors. DBS has enhanced patients’ quality of life and deepened our understanding of ETs underlying pathophysiology and electrophysiology [52]. In experimental studies, a synchronization between the tremor frequency and the Vim cell frequency was already observed, and an electrical stimulation by directional lead can cause an interruption of the tremor [53].

There is a strong recommendation for performing DBS over thalamotomy because DBS does not cause a permanent lesion, can be performed bilaterally if deemed necessary, and allows for future adjustments if these should become necessary over time [54]. There are significant side effects associated with the leads of the DBS. Still, these side effects can be minimized by adjustments to the lead and changes in electrical parameters such as current and voltage.

### 4.3. Dystonia

After pharmacological agents and botulinum toxin injection failures, surgical treatment of dystonia is recommended. The most common therapeutic target is the GPi. Noteworthy, there is a possible somatotopic organization of the GPi structure with focal and segmental dystonias. The segments of GPi are organized in hind-limb, fore-limb, and oro-facial, which can partially explain the different types of presentations of dystonia. Interestingly, the intercommissural plane was the area with the highest benefit after DBS therapy [55]. In the literature, more than fifty studies have been reported to evaluate the effects of DBS therapy on dystonia [56]. One of the most common scales for evaluating DBS therapy in dystonia is the movement subscore on the Burke-Fahn-Marsden Dystonia Rating Scale (BFMDRS) [57].

### 4.4. Obsessive-Compulsive Disorder

Obsessive-compulsive disorder (OCD) is a psychiatric condition affecting about two percent of the population, marked by chronic and persistent urges or thoughts leading to compulsive behaviors. The pathophysiology of OCD is poorly understood, but it is believed to involve the cortico-striatal-thalamic loop circuits, mainly [58]. The DBS device probably inhibits or functionally overrides this pathological network hyperactivity [59].

The humanitarian device exemption (HDE H050003) received in 2009 by DBS therapy for OCD changed the course of research regarding neurostimulation and neuropsychiatric diseases. Devices that receive HDE are usually exempt from the effectiveness requirements by the US FDA (Sections 514 and 515 of the FD&C Act), and they have specific restrictions and profit limits [60]. The approval of this neuromodulation therapy for OCD significantly changed the therapeutic options for the management of neuropsychiatric diseases.

The targets already studied in the management of OCD are the anterior limb of the internal capsule, bed nucleus of the stria terminalis, GPi, inferior thalamic peduncle, medial forebrain bundle, nucleus accumbens, ventral striatum/ventral capsule, subthalamic nucleus, and ventral striatum/ventral capsule. These treatments have yielded significant improvements in OCD symptoms (>30% reduction), encompassing obsessions, compulsions, and enhancements in social functioning [61]. Many authors only recommend DBS therapy for patients with OCD in the context of a clinical trial due to the relative lack of efficacy data and the invasive nature of the procedure [62].

One significant benefit of DBS is that throughout the OCD course, the specialist can make electrical adjustments to benefit the patient with the symptoms that most affect his life. Noteworthy OCD symptoms have a significant qualitative characteristic for the diagnosis. Also, the individual can benefit from some symptoms from neurostimulation and others from neuropharmacological therapy. 

### 4.5. Epilepsy

In approximately one of every three individuals with epilepsy, the condition does not improve with appropriate medical treatment [63]. For these drug-refractory cases, surgical options have traditionally involved resective surgery in those individuals with focal foci [64,65]. However, some individuals can have multiple foci or an unknown source; in these cases, DBS has emerged as a treatment strategy. Using leads with neurosensitivity technology, an opposite effect can be provided, inhibiting the electrical activity. Also, using closed-loop significantly improved the battery life in epilepsy, better delineating the quantity of energy that should be applied. The exploration of DBS for medically refractory epilepsy dates back to the 1970s and 1980s, initially targeting the cerebellum and the anterior nucleus of the thalamus (ANT) [66]. The anterior nucleus of the thalamus is mainly related to alertness, and its stimulation can lead to inhibition of seizure propagation. More recently, other potential DBS targets have been identified, including the centromedian-parafascicular complex (CMPfc) and the hippocampus [67].

Over the past decade, several studies have been published on the DBS of the ANT. The stimulation of the ANT for epilepsy (SANTE) trial was the first randomized controlled trial focusing on the ANT, involving 110 patients. The SANTE trial demonstrated a median 56% reduction in seizures at two years, with an increased reduction of 69% at 5 years. Based on this robust level 1 evidence, the US FDA recently approved ANT-DBS as a treatment for epilepsy [68].

While epilepsy is primarily characterized by seizure frequency, duration, and severity, patients often experience cognitive and behavioral deficits. Consequently, patients commonly undergo neuropsychological testing before considering surgical intervention [69]. Evidence suggests that DBS may improve executive function, depression, anxiety, attention, and mood, probably due to the direct control of the number of seizures. However, the precise mechanisms behind cognitive improvements require further study because they can be related to neuroplasticity [70]. Noteworthy, the ANT is associated with attention, affection, and acute memory, besides the control of alertness.

DBS typically employs continuous or “open-loop” stimulation. However, a growing interest is in developing “closed-loop” stimulation options that deliver therapy based on electrographic biomarkers [71]. With advancements in responsive neurostimulation, recent trials have indicated this personalized stimulation approach’s potential efficacy and safety [72]. Nevertheless, no direct comparisons (head-to-head trials) have been made between DBS and responsive neurostimulation for epilepsy treatment.

DBS therapy for epilepsy should be primarily assessed by the area most commonly affected by the epileptic activity. The implantable pulse generator is usually a dual channel, with one sensing electrode in the subdural area of the most common adjacent foci and another channel with a depth electrode localized in the anterior nucleus of the thalamus. The depth electrode is stimulated when the subdural electrode senses seizure activity. An interesting fact about the DBS of the ANT in epilepsy is that there was a worsening of cognition and depression even after improving seizure episodes [68].

### 4.6. Tourette Syndrome

Tourette syndrome is characterized by involuntary repetitive movements and vocalizations and is associated with disruptions in the cortico-striato-thalamo-cortical circuit. Over 150 reported cases of DBS for Tourette syndrome exist [73]. Approximately half of these patients received thalamic DBS (dorsomedial or CMPfc), while around 40% had pallidal DBS (anteromedial GPi, posteroventral GPi, or a combination). The remaining cases involved DBS of either ALIC/NAc or STN. These treatments typically resulted in a median improvement of more than 50% in the Yale Global Tic Severity Scale score, indicating a positive role in treating Tourette syndrome [74]. 

The mechanisms related to improving clinical manifestations of Tourette syndrome with DBS may be related to dopaminergic modulation, pathological oscillations, functional brain (cortical) modulatory effects, and closed-loop stimulation. In patients with Tourette syndrome, treatment with DBS can significantly increase endogenous dopamine. Also, there is a significant increase in gamma compared to alpha oscillations in DBS therapy. Noteworthy, slow frequency is commonly observed in individuals with Tourette syndrome.

### 4.7. Major Depressive Disorder

There is growing interest in extending DBS to other neuropsychiatric conditions due to evidence of network alterations. Studies evaluating DBS for depression have yielded variable efficacy. Various cortical and subcortical structures, including the subcallosal cingulate gyrus, NAc, medial forebrain bundle, VC/VS, and ITP, have been tested [75]. The largest study to date was a randomized controlled trial focusing on the DBS of the subcallosal cingulate gyrus, which did not show significant antidepressant efficacy [76]. While DBS offers promising network changes and clinical outcomes, further research is necessary to identify the ideal target and stimulation parameters.

One significant drawback of DBS therapy for depression and other neuropsychiatric disorders is the target. DBS is mainly a target therapy, in which a focus should be chosen for the therapy. In this way, diseases like major depressive disorder do not have a single anatomical location for the pathophysiology and will have lower benefits with DBS. Also, the majority of the studies that we have in the literature on neuropsychiatry conditions were only conducted for a short period of time. Thus, only long-term DBS therapy may possibly be associated with behavioral symptom control in neuropsychiatric conditions such as major depressive disorder.

### 4.8. Eating Disorders and Obesity

Morbid obesity and anorexia nervosa represent opposite ends of the weight spectrum, with defined body mass index (BMI) thresholds. Understanding the underlying biology of eating and body image perception involves complex mechanisms, including reward pathways, homeostasis, and hunger/satiety centers [77].

Several DBS trials have addressed morbid obesity by targeting motivation, volitional control, addiction, and feeding/satiety centers [78]. The lateral hypothalamus, known as the feeding center, has been explored as a potential DBS target [79]. Initial case series have suggested weight loss with lateral hypothalamic DBS, but further research is needed to confirm these findings. The nucleus accumbens (NAc) has also been investigated as a DBS target to reduce the reward associated with eating, showing some success [80]. Other theoretical targets include the medial and lateral orbitofrontal cortex, medial prefrontal cortex, ventral pallidum, caudate, insula, anterior cingulate cortex (ACC), amygdala, putamen, and hippocampus [81].

Early evidence for treating anorexia nervosa was based on lesioning procedures. Network dysfunction associated with self-awareness, visual and gustatory sensation, and the reward pathway has been identified [82]. Several DBS studies for anorexia nervosa have targeted the subgenual cingulate cortex, NAc, VC/VS, and bed nucleus of the stria terminalis, showing promising but not definitive results [83]. Further research is needed to refine DBS approaches for these complex eating disorders.

A multidisciplinary evaluation should be performed for eating disorders before choosing DBS therapy. There is a significant overlap between other neuropsychiatric conditions and eating disorders, which can explain the different ranges of improvement in the symptoms of individuals. It is possible that a better approach would be dual management with neuropharmacological and neuromodulation simultaneously to control the neuropsychiatric comorbidities and the eating disorders, respectively.

### 4.9. Substance Abuse/Addiction

Addiction and substance abuse are significant social issues, and DBS has been explored as a potential treatment. The primary DBS targets for addiction are related to the nucleus accumbens (NAc) and the subthalamic nucleus (STN) [84]. In this context, the main idea of the DBS is to decrease drug-seeking behavior. The NAc was commonly targeted because it is a fundamental structure in the mesolimbic reward pathway. Also, the decreased functioning of the prefrontal cortex in functional studies was already related to addiction behavior. Interestingly, it is believed that chronic substance use can lead to neuroplasticity in individuals with addiction. So, the beneficial outcomes of DBS would be seen after long-term therapy due to a possible reversal or change in the brain’s cytoarchitecture [84]. 

Studies have often focused on NAc and STN due to the side effects observed in DBS treatments for other disorders, such as OCD. Given the complexity of psychiatric disorders like addiction, there have been investigations into modulating multiple nodes in the circuit simultaneously. For example, some studies have evaluated concomitant targets like the NAc and anterior limb of the internal capsule (ALIC) [85].

### 4.10. Chronic Pain

Chronic pain is a prevalent condition that affects a significant portion of the population. Various categories of chronic pain exist, including nociceptive, deafferent, central, and peripheral pain. The use of neuromodulation, including DBS, for chronic pain dates back to the 1950s. Earlier techniques for treating intractable pain involved lesioning procedures. Cingulotomy, a procedure targeting the anterior cingulate cortex (ACC), hinted at the potential of the ACC as a DBS target for pain relief [86].

**Figure 3:**
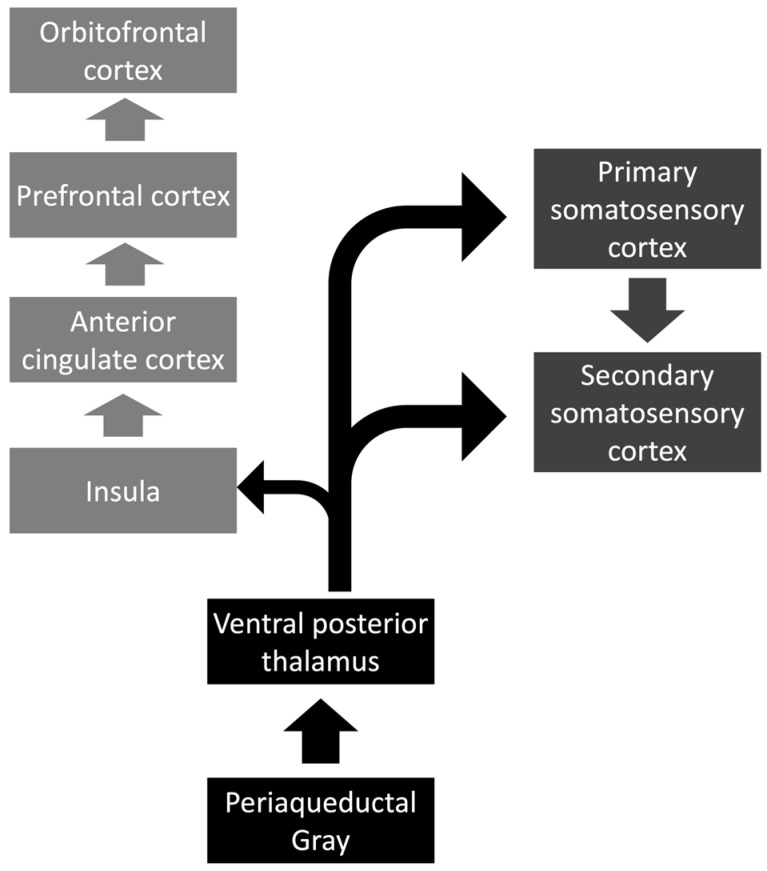
The main targets of deep brain stimulation for the management of chronic pain.

Three main targets have already been studied for managing chronic pain with DBS. First, the periaqueductal and periventricular gray (PAG/PAV) are related to the production of enkephalin, which can inhibit pain. Therefore, stimulating this area leads to the release of enkephalin, inhibiting the transmission of pain to cortical structures. Another area that was commonly studied was the sensory thalamus, especially the ventral posterior lateral nucleus/ventral posterior medial nucleus (VPL/VPM), a main structure related to the two most common pathways related to the transmission of pain. In this way, the main mechanism for pain inhibition may be inhibiting the VPL/VPM structures. The third structure is the anterior cingulate gyrus, commonly related to the emotional feelings associated with chronic pain. Noteworthy, these pathways are related to pain sensitivity, so their stimulation is believed to lead to inhibition of the conduction (Figure 3). Other potential targets include the CMPfc, the ventral striatum/anterior limb of the internal capsule (VS/ALIC), and the posterior hypothalamus [87].

Some studies have also investigated combined PAG/PVG and VPL/VPM stimulation, although the results have been inconclusive. Motor cortex stimulation has been explored as an alternative approach for various types of chronic pain, including facial neuropathic pain, phantom limb pain, postherpetic neuralgia, brachial plexus avulsion, Wallenberg syndrome, complex regional pain syndrome, multiple sclerosis-derived pain, spinal cord injury pain, and post-traumatic brain injury pain [87,88]. A significant concern in targeting the cortex is the fact that there is a susceptibility to increased cortical activity and the development of epileptic activity.

### 4.11. Alzheimer’s Disease

Pharmacological therapies for Alzheimer’s disease have shown limited success, prompting the investigation of alternative approaches such as gene therapy and DBS [89]. Recent research has revealed disruption in the memory network functions, including the Papez circuit, default mode network, and salience network, suggesting that neuromodulation of these networks may improve cognition impairment [90]. DBS was also studied in other cognitive disorders such as mild cognitive decline, dementia with Lewy bodies, frontotemporal dementia, and Parkinson’s disease dementia. It is worth noting that the fornix, a white matter tract within the Papez circuit, plays a vital role in memory and cognition. Phase 1 and 2 clinical trials have targeted the fornix with DBS therapy for managing Alzheimer’s disease [91].

Half of a phase 1 trial involving six patients exhibited a statistically significant improvement in the cognitive subscore on the Alzheimer’s disease assessment scale (ADAS-Cog). In contrast, the other half displayed mild improvements in ADAS-Cog scores. Based on these results, a phase 2 double-blind, randomized controlled trial for Fornix DBS was conducted. However, no statistical difference was found between the groups with and without DBS [91]. Interestingly, older individuals presented better outcomes in the sensitivity analysis. In this way, patients over 65, compared to those under 65 years old, experienced less decline in the ADAS-Cog and the Clinical Dementia Rating Scale Sum of Boxes. A phase 3 clinical trial is being developed to assess the effects of fornix DBS in patients over 65. The main mechanism for improving fornix DBS is believed to occur due to direct activation of the cognition-related neural circuits. Some authors believe that this benefit is only temporary because the patient may have improved due to increased attention. Therefore, future studies related to cognitive impairment and dementia should specifically evaluate all aspects of cognition with extensive neuropsychological tests using a prospective assessment method. One year after continuous stimulation, memory networks (fronto-temporo-parieto-striato-thalamic and fronto-temporo-parieto-occipito-hippocampal networks) and the default mode network showed increased activation [92].

The cholinergic neuronal system is associated with learning and some aspects of the memory process. However, in patients with AD, the cholinergic neurons are the most frequently affected structures. One of the main structures related to the cholinergic system is the nucleus basalis of Meynert (nbM), which is commonly affected in neurodegenerative diseases [93]. Therefore, the stimulation of this structure is believed to promote the local production of acetylcholine and the electrode functioning as part of the cholinergic system that was already completely damaged. In a pilot study, six patients underwent bilateral nbM DBS for AD, with four showing positive responses regarding stable Mini-Mental State Examination and ADAS-Cog scores. AD often leads to executive function decline. The ventral capsule/ventral striatum (VC/VS) is part of neural networks associated with executive function, including the dorsomedial and orbitofrontal cortices [94]. A pilot trial involving three patients receiving VC/VS DBS demonstrated less cognitive decline than age-matched controls. Moreover, VC/VS DBS resulted in frontal cortical activation [95]. Both nbM and VC/VS DBS hold promise as safe and potentially effective treatment strategies but require further supportive evidence.

The main targets of DBS for the management of AD are localized structures. However, AD is a neurodegenerative disease that affects a significant number of structures at the same time. In this way, the local stimulation of a structure can lead to a partial improvement of the cognitive function of the individuals, which will depend on the structures mainly affected by the individuals. Therefore, techniques that could better delineate the main area affected using functional neuroimaging should be conducted before the DBS procedure.

### 4.12. Posttraumatic Stress Disorder and Anxiety Disorder

Posttraumatic stress disorder (PTSD) can affect various cognitive and psychiatric domains [96]. Disruptions in multiple brain networks, including the default mode network, salience network, ventral attention network, and affective network, have been observed in individuals with PTSD. As a result, various forms of neuromodulation have been explored as potential treatments, including electroconvulsive therapy, transcranial magnetic stimulation, vagal nerve stimulation, and DBS. 

DBS for PTSD has targeted brain regions such as the basolateral amygdala and, theoretically, the subgenual anterior cingulate gyrus. Additionally, there has been interest in using DBS for anxiety based on improvements observed during DBS of the anterior limb of the internal capsule (ALIC) for OCD [97]. However, there are descriptions of cases involving DBS of the nucleus accumbens (NAc) for panic disorder, worsening the neuropsychiatric disorder [98].

The most common limitations of DBS for managing PTSD overlap those related to major depressive disorder, addiction, and OCD. The neuropsychiatry conditions do not have a single, straight pathway to explain. Also, it is believed that they may have several modulators. Therefore, the most effective management of these disorders could be the therapeutic combination of neuropharmacology and neuromodulation.

### 4.13. Tinnitus

The conscious perception of auditory sensations without external stimuli has also been a subject of neuromodulation research. The auditory cortex and limbic pathways are believed to play a role in tinnitus pathophysiology. Notably, the main area related to the auditory cortex is the superior temporal lobe bilaterally. Therefore, a more precise anatomical localization could be estimated compared to neuropsychiatric diseases.

Transcranial magnetic stimulation over the temporoparietal cortex has been used to suppress excitability and treat tinnitus. DBS targeting Heschl’s gyrus has been explored as a potential treatment for tinnitus. Several brain regions, including the Vim, locus of the caudate neurons (area LC), STN, amygdala, and hippocampus, have been suggested to modulate tinnitus. Some case reports have shown that patients receiving DBS for movement disorders experienced concurrent improvements in their comorbid tinnitus with STN and Vim stimulation [99]. Intraoperative evidence has also indicated tinnitus reduction when an electrode lead passes through area LC, highlighting its importance in tinnitus research [100]. Another pathway that can be targeted in the management of tinnitus with DBS is the medial geniculate body, but there are significant side effects related to the procedures in this area.

### 4.14. Refractory Aggression

Aggressiveness, characterized by various intensity attack patterns such as physical, verbal, facial, indirect, and sexual attacks, is typically regulated by the limbic system and is often considered innate. Pathological aggressiveness occurs when the response to triggering stimuli is disproportionate and magnified, leading to self-inflicted harm or harm to others, making it a critical issue to address in psychiatric patients [101].

Traditionally, pathological aggressiveness has been managed through psychiatric interventions, including drug therapy. However, some patients are refractory to therapy or experience adverse metabolic effects from medications.

In a study by Franzini et al. [102], DBS was explored as a treatment for aggressive and disruptive behavior refractory to conservative approaches. This study involved stereotactic methodology and was performed under general anesthesia. Seven patients received bilateral DBS of the posterior hypothalamic region, recorded with neurosensitive devices with microrecording activity. The authors found that more than 85 percent of the patients experienced a significant reduction in aggressive behavior. This suggests that DBS may offer an alternative and beneficial approach for managing pathological aggressiveness in cases where traditional treatments prove ineffective or have significant side effects [102].

### 4.15. Bipolar Disorder

The data regarding DBS in bipolar disease are scarce and limited in quality. One common DBS target is the subcallosal cingulate gyrus and the ventral capsule/ventral striatum [103]. However, these anatomical sites were commonly associated with adverse effects such as hypomanic symptoms, which resolved after the discontinuation of the stimulation. The largest study was a 24-week prospective observational trial in which seven patients with bipolar major depression received add-on treatment with DBS that targeted the subcallosal cingulate gyrus. The authors observed a significant improvement of the baseline depressive symptoms by approximately 45 percent, with functional improvement. Also, the beneficial outcomes of DBS for depressive symptoms were persistent in the long-term follow-up two years after DBS implantation [104].

### 4.16. Headache

There are no strict indications for DBS in individuals with headaches. The literature is scarce regarding this subject. The most common types of headaches studied are cluster headaches and short-lasting unilateral neuralgiform headache attacks. In an analysis of individual patient data of forty individuals with cluster headaches, DBS was associated with a 77 percent mean reduction of headache frequency among 75 percent of responders [105]. The two main DBS targets for headache management are the posterior inferior hypothalamic region and the midbrain tegmentum [106].

### 4.17. Chorea

In the literature, case reports showed the benefits of DBS in choreiform disorders. High- and low-frequency stimulation were reported, but the higher frequency was associated with significant side effects. The frequency applied for Huntington’s disease was 40 to 180 Hz, and the frequency for neuroacanthocytosis was 40 to 160 Hz. Also, only half of the cases used the microelectrode recording technique. The patients improved their choreiform symptoms, but there was a worsening of dysarthria, hypotonia, and gait issues. The most common target of DBS was GPi, which was based on previous surgical lesioning procedures [107].

### 4.18. Restless Legs Syndrome

The discovery of DBSs efficacy in managing restless leg syndrome was an indirect finding in studies of DBS for PD. Klepitskaya et al. found that targeting STN for patients with PD significantly improved the International RLS Study Group Rating Scale. After two years, three of every four patients with PD had at least fifty percent improvement in their restless leg symptoms [108]. Interestingly, GPi could also be a target for managing refractory restless legs syndrome. In a case report targeting GPi, the patient had significant improvement in his reported symptoms, and the polysomnography findings suggest an independent factor of efficacy [109].

## 5. DBS Target

One important step in the DBS procedure is anatomical target selection. There is no universal consensus on the ideal target for DBS for most neurological conditions. Also, drug-resistant individuals may not have uniform features that could be corrected with the same approach. Even though they have a similar diagnostic definition, there may be different pathophysiological explanations for their medication resistance. In this context, the response will not be the same for all individuals with a drug-resistant neurological condition. Table 6 shows the most common targets of DBS (Table 6).

## 6. Efficacy and Clinical Outcomes of DBS Therapy for Movement Disorders

When considering DBS surgery for patients with PD, physicians, patients, and caregivers must be aware of the expected outcomes of motor symptoms. On average, PD patients can expect an improvement of about 50% in their Unified Parkinson’s Disease Rating Scale part III (UPDRS III) scores six months to two years after DBS surgery [126]. Specifically, one year after DBS, tremors tend to improve by approximately 74%, rigidity by 57%, and bradykinesia by 49%. Dyskinesias are expected to significantly improve, with a mean reduction of 80% in duration and 94% in disability one year after DBS. Axial symptoms, such as posture and gait, may show an initial improvement of up to 57% during the first year. Still, the motor symptoms could worsen by about 6% after eight years due to the ongoing progression of the underlying disease [127].

Some studies have reported sustained benefits of DBS for several years, suggesting its long-term efficacy. Many patients experience improved quality of life, reduced medication use, and better motor control after DBS. The efficacy and clinical outcomes of various movement disorders will be further discussed.

### 6.1. Dystonia

In comparing DBS targeting the globus pallidus internus (GPi) and the subthalamic nucleus (STN) for treating dystonia, 35 studies were analyzed with 319 GPi-DBS and 113 STN-DBS patients. In the average follow-up of 12.48 months, both GPi and STN groups showed similar efficacy, improved quality of life, mood, and the occurrence of adverse effects. However, patients with focal dystonia experienced better disability symptom improvement than segmental and generalized cases, though their quality-of-life improvement was lower than the segmental group. Primary dystonia cases responded better to DBS than secondary dystonia, with fewer adverse effects. Importantly, improved movement symptoms correlated with better disability symptom improvement. In conclusion, both GPi-DBS and STN-DBS are safe and effective in enhancing the quality of life and reducing dystonia symptoms in patients. Patients with different dystonia types and etiologies may have varying responses to DBS, with focal and primary dystonia cases showing more favorable outcomes [128].

### 6.2. Parkinson’s Disease

A meta-analysis compared the long-term efficacy of subthalamic nucleus (STN) and globus pallidus interna (GPi) DBS for PD. The analysis found that STN-DBS and GPi-DBS significantly improved PD patients’ motor function and daily living activities. Specifically, the Unified Parkinson Disease Rating Scale (UPDRS) III scores during the off-medication state showed no substantial difference between the two DBS targets, indicating similar long-term efficacy in managing motor symptoms [129]. Subgroup analysis demonstrated stability in UPDRS III scores from baseline to 2 years post-DBS, with slight improvement at three years, though not statistically significant. Tremor, rigidity, and gait scores positively changed with both DBS targets. Additionally, patients experienced a significantly improved quality of life as measured by the Parkinson’s Disease Questionnaire (PDQ-39 ADL) and a notable reduction in levodopa-equivalent dosage after DBS. In summary, STN-DBS and GPi-DBS effectively enhance motor function and daily activities in PD patients, with no significant difference in long-term motor symptom management. These findings support both as viable DBS treatment options for PD [130].

### 6.3. Wilson’s Disease

According to a recent report, a 29-year-old patient with severe generalized dystonia caused by Wilson’s disease underwent bilateral DBS to evaluate clinical effectiveness. The Burke–Fahn–Marsden Dystonia Rating Scale was used for the primary assessment, followed by the Abnormal Involuntary Movement Scale and the Zarit Caregiver Burden Interview score, all measured 20 weeks (approximately four and a half months) after the surgical procedure [131].

Results indicated a 14% improvement in the Burke–Fahn–Marsden dystonia scale motor severity score, signifying a positive impact on dystonia symptoms. However, there was no significant change in the abnormal involuntary movement scale score. The Zarit caregiver burden interview scale exhibited a substantial 44.4% improvement, suggesting a reduction in caregiver burden.

In summary, bilateral DBS in the globus pallidus internus can effectively alleviate dystonia and caregiver burden in patients with Wilson’s disease. The outcomes may vary based on the disease stage during the surgical procedure. This study underscores the potential benefits of this intervention in managing Wilson’s disease-related dystonia.

## 7. Safety and Adverse Effects of DBS Therapy

DBS is generally considered a safe and effective treatment for various neurological conditions. However, like any medical procedure, it has potential risks and adverse effects. The location of electrode implantation played a significant role in determining the side effects (Table 7).

Tiredness/fatigue were commonly associated with DBS in the thalamus. Affective side effects, such as depression or suicidal ideation, often follow implantations in the subthalamic nucleus. Higher voltage settings of the electrode were linked to more severe depression following implantation [132].

Immediate complications after DBS surgery include intracranial hemorrhage, which can occur in 4.4% of cases but is reduced to 0.7% when performed by experienced medical centers. The risk of symptomatic bleeding leading to permanent deficits is approximately 1.6%. Infection is the most common surgery-related complication and affects more than five percent of individuals. In the postoperative period, weight gain is a prevalent adverse event, occurring in 36% of patients. This weight gain may result from various factors, including improved control of dyskinesias and behavioral changes. Another significant side effect is a change in speech intelligibility, characterized by dysarthria and hypophonia, which can be observed in approximately 20% of patients. Notably, these speech changes after DBS can be attributed to the surgery (possibly due to adverse lesional effects), disease progression (unrelated to DBS), or the spread of electrical currents affecting speech-related areas. Adjustments in DBS programming may partially address these concerns [127,133].

DBS surgery is generally considered safe from a cognitive perspective when patients are carefully assessed before the procedure following a comprehensive preoperative evaluation protocol. However, despite the safety measures and technological advances, some symptoms in DBS for conditions such as PD remain unresponsive [134]. Challenges include limitations in predictive factors such as the levodopa challenge test, the resistance of certain symptoms, the need for new targets, and obstacles in implementing technologies such as BrainSense for closed-loop stimulation. Surgical planning and limited access to remote DBS programming also challenge optimizing the therapy [127].

## 8. Programming and Follow-Up

Over the past decade, DBS has seen significant technological advances. Notable improvements include IPG design, which is smaller, rounded-edge implanted pulse generators (IPGs) that reduce the risk of complications. Patients can choose between rechargeable and non-rechargeable options and MRI-compatible devices.

Directional electrodes enhance targeting accuracy by allowing subtle adjustments to avoid stimulating adjacent structures. Multiple independent current controls enable precise current regulation at different electrode contacts, enhancing efficiency. New brain-sensing devices record local field potentials, aiding in PD symptom monitoring. Beta bursts (13–30 Hz) correlate with motor symptoms, while gamma bursts (30–200 Hz) relate to dyskinesias. A recent study by Muthuraman et al. discovered that DBS can effectively reduce beta power and increase gamma power in brain regions connected to the basal ganglia. This study also observed cross-frequency coupling of gamma oscillations in the cortico-basal ganglia brain network during clinically effective deep brain stimulation, which is worth noting due to its negative correlation with motor deficits. This study emphasizes the importance of gamma oscillations in improving the motor deficits associated with Parkinson’s disease [135]. This study emphasizes the importance of gamma oscillations in improving the motor deficits associated with Parkinson’s disease [127].

In studies with a follow-up duration of five years or more, a comprehensive assessment of adverse events revealed a range of permanent and transient effects associated with the medical intervention under investigation. Weight gain was the most prevalent among the permanent adverse effects, affecting 90 patients [136]. Dysarthria, characterized by speech difficulties, was reported in 50 patients, while eyelid apraxia, leading to an inability to open or close the eyelids voluntarily, was observed in 38 patients. Regrettably, 22 patients experienced permanent adverse effects resulting in death, four of which were attributed to suicide. Additionally, 22 patients exhibited apathy, 20 experienced cognitive decline or dementia, 14 were affected by depression, 10 had dyskinesia (involuntary movements), and nine had dystonia (abnormal muscle contractions). Fewer patients reported permanent effects such as symptomatic hemorrhage (3 cases) and mania or hypomania (2 cases) [36,137].

In contrast to the permanent adverse effects, transient effects were not enduring. Confusion was reported in 25 patients, while depression was experienced by 23 patients. Infection and/or erosion related to the implanted device affected 13 patients. Mania was observed in 11 patients, apathy in 10, psychosis in 9, and seizures in 5 patients. These findings highlight the necessity of a thorough assessment, monitoring, and management of potential adverse effects in medical interventions, especially when considering treatments with extended follow-up periods of five years or more [137].

DBS is a growing therapeutic method for neurological disorders. It involves implanting hardware in the brain and subcutaneous tissue. Common hardware complications of DBS include electrode migrations or misplacements, fractures of the extracranial electrode portion, infections, erosions, and implantable pulse generators [138]. The reported complication rate per electrode year ranges from 3.2% to 8.4%. In a study involving 215 patients with various movement disorders treated with DBS, 23.2% visited the emergency room due to DBS-related issues. These visits were primarily prompted by neurological symptoms (54.6%) and infections or hardware complications (27.9%). Patients with PD tended to experience more early complications within the first six months post-surgery, while patients with dystonia were more prone to late complications occurring beyond six months after the procedure [139].

## 9. Conclusions

Several neurological conditions resistant to medication can be successfully treated with DBS. Even though the underlying mechanics are not fully understood, DBS has greatly advanced our knowledge of human physiology. The US FDA has only approved the use of DBS to treat Parkinson’s disease, essential tremor, dystonia, and OCD, even though many conditions have already been studied. Also, most of them have shown the cost-benefit of DBS procedures through numerous studies. Future research should assess the significance of DBS in neurogenesis and plasticity processes and methods to improve these effects. Currently, DBS is only used for the symptomatic management of neuropsychiatric disorders.

## Figures and Tables

**Table 1:** Timeline of the deep brain stimulation development.

Year	Description
1874	Electrical stimulation of the human cortex was performed by American physician Robert Bartholow
1947	The stereotactic frame was developed for human neurosurgery. Ernest A. Spiegel developed a stereotactic frame, which was followed in 1949 by the arc-based Leksell frame
1948	J. Lawrence Pool performed the first chronic DBS implantation using an electrode connected to an induction coil
1952	The first stereotactic atlas with coronal photographs of the brain was published
1954	Acute thalamic DBS to target chronic pain. It is considered one of the first functional applications for DBS
	Acute DBS used in pre-lesion targeting for psychiatric disorder
1958	The first definitive cardiac pacemaker was implanted. The first temporary transcutaneous cardiac pacing device was made in 1952
1960	Acute DBS is used to identify lesion targets in essential tremor
	Frequency-dependent effects of DBS reported
1961	The first human intraoperative microelectrode recordings
1963	José Manuel Rodríguez Delgado used a “stimoceiver” to inhibit the aggressive behavior of a bull
1968	Medtronic implantable pulse generator. Also, the first spinal cord stimulator was commercially available
1970s	Computed tomography is used for stereotactic targeting
	Radiofrequency control on an “external” transmitter on DBS systems
1972	The first chronic DBS implant for PD
1973	Thalamic DBS for denervation pain
1977	Periventricular DBS for pain
1980s	MRI is used for stereotactic targeting
	The first fully intracranial DBS devices were available. Also, the long-lasting implantable lithium batteries greatly extend implant life and the maintenance of the device
1980	DBS for multiple sclerosis tremor
1987	DBS of the ventral intermediate nucleus of the thalamus was effective in the management of tremor in individuals with PD
	DBS therapy for the management of tremors was successfully reported by Alim-Louis Benabid
1990	Refinement of battery-driven pacemakers
	DBS reverses motor symptoms in MPTP-induced parkinsonism in monkeys
1994	DBS of the subthalamic nucleus is used in the management of tremors in patients with PD
1997	The FDA approves DBS of the ventral intermediate nucleus of the thalamus for the management of essential tremors
1999	DBS of the anterior limb of the internal capsule was first used to manage obsessive-compulsive disorder
	Visser-Vanderwalle reported the effective use of DBS of the medial thalamus in three patients with Tourette’s syndrome
	Globus pallidus internus DBS for the management of refractory dystonia
	Implantable pulse generators with dual-channel technology, which was developed after the creation of dual chamber cardiac pacing in 1998
2000s	DBS therapy is refined for treating essential tremors, PD, and dystonia
2002	US FDA approves DBS in PD
	Quadripolar electrodes are commercially available
2003	The US FDA approves DBS for dystonia
2004	Computer models of DBS
2005	DBS is used to treat depression
2007	DBS is used to treat minimally conscious states
2009	DBS of the bilateral anterior limb of the internal capsule for the management of obsessive-compulsive disorder received a humanitarian device exemption from the FDA
	Rechargeable DBS batteries are available
2010	Sin Alzheimer’s pilot trial evaluates the DBS of the fornix
2011	Close-loop stimulation for epilepsy management
2013	DBS device capable of simultaneous stimulation and recording activities of the local field potential signal processing.
	DBS of the subcallosal cingulate gyrus in an anorexia pilot trial
	A closed-loop, responsive DBS system was introduced to treat epilepsy. These devices need to have neural activity sensitivity, leading to a decreased number of side effects and a longer battery life
2015	The emergence of directional DBS leads can lead to an adjustment of the electrical field along the lead axis
2018	The US FDA has approved DBS as an add-on treatment for drug-resistant epilepsy in adults
2020	The US FDA approves a DBS device capable of neurosensitivity and directional leads
	Wireless devices with three Tesla MRI compatibility

Abbreviations: DBS, deep brain stimulation; MRI, magnetic resonance imaging; PD, Parkinson’s disease; US FDA, US Food and Drug Administration.

**Table 2:** Stimulus-induced side effects in DBS surgical procedures.

Adverse Event	DBS Target	Region Related to the Side Effect	Correctional	Reference
Dyskinesias	GPe, GPi, STN	Excessive modulation of the indirect pathway	Decrease frequency. Removal of leads	[17]
Dysphonia, dysarthria	STN, GPi	Internal capsule and associate circuits of basal ganglia	If possible, change the hemisphere	[18]
Muscle contractions	STN, GPi, VOP	Corticospinal tract of the internal capsule	Move posterior	[19]
Mood changes, risky behavior	GPi, STN	Associative and limbic circuits of the basal ganglia	Move dorsal	[20]
Oculomotor disturbances	GPi, STN	Internal capsule for conjugate eye deviation Third nerve medial to STN for ipsilateral eye movements	Move medial; Move lateral	[21]
Paresthesia	Vim, STN, VOP, PPN	Lemniscal fibers	Move anterior	[22]
Phosphenes	GPi	Optic tract	Move medial	[23]
Sadness, depression	STN	Ventromedial STN, substantia nigra pars reticularis	Move dorsal	[24]
Verbal fluency, working memory	GPi, STN	Associative circuits of the basal ganglia	Move dorsal	[25]
Weight gain	STN, GPi	Normalization of energy metabolism	Increase physical activity	[26]

Abbreviations: DBS, deep brain stimulation; GPe, globus pallidus externus; GPi, globus pallidus internus; PPN, pedunculopontine nucleus; STN, subthalamic nucleus; Vim, ventral intermediate nucleus of the thalamus; VOP, posterior ventral oral nucleus.

**Table 3:** Features of implantable pulse generators for deep brain stimulation.

Model	No. of Chambers	Weight (g)	Size (mm)	Rechargeable Cell	Frequency Range (Hz)	Pulse Width (μs)	Temporal Fractionation	Current Fractionation	Directional Lead	Magnetic Resonance Safety	Local Field Potential
St. Jude (Abbott) Infinity 5 ^a^	2	49	56 × 50 × 13	No	2–240	20–500	Multi-stim set	Coactivation	Yes	Conditional: more than 1.5T requires specific conditions	No
St. Jude (Abbott) Infinity 7 ^a^	2	58	67 × 50 × 14	No	2–240	20–500	Multi-stim set	Coactivation	Yes	Conditional: more than 1.5T requires specific conditions	No
Boston Scientific Vercise PC ^b^	2	55	71 × 50 × 11	No	2–255	10–450	Areas	Multiple independent current controls	Yes	Unsafe failure of the equipment	No
Boston Scientific Vercise Gevia ^b^	2	26	51 × 46 × 11	Yes	2–255	20–450	Areas	Multiple independent current controls	Yes	Conditional	No
Boston Scientific Vercise Genus P8/P16 ^b^	1 or 2	58	72 × 50 × 12	No	2–255	20–450	Areas	Multiple independent current controls	Yes	Conditional	No
Boston Scientific Vercise Genus R16 ^b^	2	27	52 × 46 × 11	Yes	2–255	20–450	Areas	Multiple independent current controls	Yes	Conditional	No
Medtronic Activa PC ^c^	2	67	65 × 49 × 15	No	2–250	60–450	Interleaving	No	No	Conditional, certain requirements	No
Medtronic Activa RC ^c^	2	40	54 × 54 × 9	Yes	2–250	60–450	Interleaving	No	No	Conditional, 1.5T MRI	No
Medtronic Activa SC ^c^	1	44	55 × 60 × 11	No	3–250	60–450	Interleaving	No	No	Conditional, but not eligible for full-body MRI	No
Medtronic Perpcept PC ^c^	2	61	68 × 51 × 12	No	2–250	20–450	Interleaving	No	No	Conditional, 3T, and 1.5T MRI	Yes

^a^ https://www.neuromodulation.abbott/us/en/parkinsons/infinity-for-deep-brain-stimulation.html, accessed on 15 October 2023. ^b^ https://www.bostonscientific.com/en-US/products/deep-brain-stimulation-systems.html, accessed on 15 October 2023. ^c^  https://www.medtronic.com/us-en/patients/treatments-therapies/deep-brain-stimulation-parkinsons-disease/about-dbs-therapy/dbs-products.html, accessed on 15 October 2023.

**Table 4:** Clinical trials of deep brain stimulation.

Condition	Identifier Number
Abdominal pain, functional abdominal pain	NCT03558009
Action tremor	NCT03156517, NCT02960243, NCT02087046
Addiction, methamphetamine, and nicotine use disorders	NCT01274988, NCT01245075, NCT01658592, NCT03424616, NCT03952455, NCT02892851, NCT05558358, NCT04432064, NCT02594306, NCT03347474
Alcohol use disorder	NCT05522751, NCT03660124, NCT05786872, NCT05884619, NCT01798888
Alzheimer disease	NCT03622905, NCT01094145, NCT00658125, NCT03115814, NCT04856072, NCT01608061, NCT03352739, NCT03290274, NCT05882344, NCT03959124, NCT00888056, NCT01559220, NCT03347084
Anorexia nervosa	NCT01678014, NCT05245643, NCT03168893, NCT01476540, NCT02593695, NCT01924598
Autism spectrum disorder, self-injurious behavior	NCT03982888
Binge eating disorder	NCT02868619
Bipolar disorder	NCT01372722, NCT01476527
Central post-stroke pain	NCT05708729, NCT05204472
Cerebellar ataxia	NCT03341416
Chronic and severe post-coma disorders of consciousness	NCT01718249, NCT02667899, NCT04502550
Chronic pain, low back pain, refractory neuropathic pain, and neuralgia	NCT04085406, NCT01072656, NCT06019793, NCT03029884, NCT03399942, NCT01899170, NCT05404581, NCT04151043, NCT02006433, NCT05451251
Cluster headache	NCT00662935, NCT02782533, NCT05857098
Cognitive impairment, mild cognitive impairment	NCT02763397, NCT00947934, NCT05417555, NCT04696978, NCT04279548
Dementia with Lewy bodies	NCT02263937, NCT01340001
Depressive disorder, major depressive disorder	NCT00837486, NCT01834560, NCT01069952, NCT00122031, NCT00555698, NCT01331330, NCT05716555, NCT01983904, NCT01095263, NCT01778790, NCT03437928, NCT00296920, NCT01921543, NCT00367003, NCT04004169, NCT01569711, NCT04530942, NCT01973478, NCT02046330, NCT05773755, NCT04106466, NCT01268137, NCT01984710, NCT02889250, NCT01435148, NCT00565617, NCT00531726, NCT04021823, NCT04009928, NCT01801319, NCT03360942, NCT01898429, NCT05418894, NCT03952962, NCT03667872, NCT03347487, NCT01798407, NCT03254017, NCT03653858, NCT05777876
Dyskinetic cerebral palsy	NCT02097693
Dystonia, cervical, generalized	NCT05416905, NCT05715138, NCT04432285, NCT00132990, NCT01671527, NCT00148889, NCT00132340, NCT00169338, NCT00142259, NCT00971854, NCT03017586, NCT02686125, NCT00580658, NCT00004421, NCT02263417, NCT00773604, NCT03078816, NCT00169403, NCT02583074, NCT04650958, NCT04568681, NCT03409120, NCT02468843, NCT05097001, NCT02982304, NCT02911103, NCT00105430, NCT00331669, NCT05870020, NCT01435681, NCT02509338, NCT02877836, NCT05506085, NCT05150093, NCT04618887, NCT04810325, NCT01497639, NCT02552628
Epilepsy	NCT03900468, NCT00772421, NCT01141764, NCT00101933, NCT03870308, NCT04692701, NCT00736424, NCT00194870, NCT04181229, NCT04164056, NCT04897776, NCT03819738, NCT00228371, NCT05437393, NCT03465163, NCT05600738, NCT05327387, NCT01210781
Essential tremor	NCT03795935, NCT02491554, NCT01334814, NCT02264925, NCT03832712, NCT00906412, NCT05096572, NCT05177900, NCT05671848, NCT05362448, NCT03984643, NCT02678429, NCT05795218, NCT02418858, NCT00634478, NCT03051178, NCT03875404, NCT05909839, NCT04260971, NCT03760406, NCT04758624 NCT04828798, NCT04032470, NCT05976074, NCT03778060, NCT04581941, NCT04212780, NCT02443181, NCT03696420, NCT02947841, NCT03769961, NCT05968976, NCT05381688, NCT03305588, NCT03811405, NCT03794661, NCT01705301, NCT05897775
Frontotemporal dementia	NCT05699330
Hypothalamic obesity due to craniopharyngioma	NCT03708913
Huntington’s Disease	NCT02535884, NCT04244513, NCT00902889, NCT02263430
Impulsive behavior	NCT04811807
Mania disorder	NCT05444907
Metabolic disorder, obesity	NCT02440945, NCT03279432, NCT02232919, NCT04453020, NCT01512134, NCT02254395, NCT01933113, NCT03650309
Multiple systems atrophy	NCT05197816, NCT04617873, NCT03593512
Multiple sclerosis	NCT00954421, NCT04062331
Neurogenic bladder	NCT03202251
Obsessive-compulsive disorder	NCT03184454, NCT01135745, NCT00169377, NCT02398318, NCT02601677, NCT00724490, NCT01879254, NCT05160129, NCT01506206, NCT01485263, NCT01985815, NCT02377375, NCT02590445, NCT03457675, NCT00057603, NCT00640133, NCT04919785, NCT01061983, NCT01329133, NCT05995951, NCT04967560, NCT01429558, NCT02844049, NCT03244852, NCT04217408, NCT05623306, NCT04806516, NCT02655926, NCT03894397, NCT01807403, NCT03463590, NCT04958096, NCT03217123, NCT04281134, NCT05915741, NCT02537795, NCT03605316, NCT02773082, NCT05422469, NCT04228744, NCT02685280, NCT05577598
Opioid-related disorder	NCT02440152, NCT02282072, NCT03950492, NCT05903495, NCT04354077
Parkinson’s disease	NCT02154724, NCT04361955, NCT05774041, NCT04725773, NCT05962489, NCT05193825, NCT02982512, NCT02937727, NCT00663312, NCT01221948, NCT00985517, NCT00664157, NCT01883973, NCT03884231, NCT04071847, NCT00355927, NCT05089682, and others. The approximate number of clinical trials with Parkinson’s disease and DBS is 300.
Parkinson’s disease and dementia	NCT01701544
Persistent development stutters	NCT05641701
Postoperative delirium	NCT05197439
Post-traumatic stress disorder	NCT01658748, NCT03416894, NCT02091843
Prader-Willi syndrome	NCT02297022
Refractory epilepsy	NCT04771065, NCT01521754, NCT05292183, NCT04753983, NCT02602899, NCT03404128, NCT05493722
Refractory schizophrenia	NCT02377505, NCT05694000, NCT02361554, NCT05337904
Spasmodic dysphonia	NCT04938154, NCT02558634
Spinal cord injury	NCT04144972, NCT04325165, NCT03053791, NCT00959296
Stroke sequelae	NCT02835443, NCT05968248, NCT05701280
Tardive dyskinesia	NCT02524886
Temporal-lobe epilepsy	NCT00717431
Tinnitus	NCT04296097, NCT03976908, NCT01988688
Tourette syndrome	NCT00478842, NCT04449068, NCT00311909, NCT04342754, NCT02056873, NCT01817517, NCT03958617, NCT01647269, NCT05371041, NCT02112253, NCT02619084
Tremor refractory to medical therapy	NCT02095600, NCT02585583

**Table 5:** Targets of deep brain stimulation for the treatment of Parkinson’s disease.

Target	STN	GPi	Vim
Tremors	++	+	+++
Bradykinesia/rigidity	++	++	-
Reduction of the dose of dopaminergic therapy	++	-	-
Reduction of dyskinesias	+	++	-
Worsening of cognitive function after DBS therapy	+++	+	+
Worsening of speech after DBS therapy	+	+	++
Occurrence of bleeding after surgery	+	+++	+

Abbreviations: DBS, deep brain stimulation; GPi, globus pallidus internus; STN, subthalamic nucleus; Vim, ventral intermediate nucleus of the thalamus; -, unlikely; +, possible; ++, likely; +++, certain.

**Table 6:** Targets of deep brain stimulation for the treatment of various disorders.

Indication	DBS Target	Reference
Addiction	Nucleus accumbens	[110]
Aggressive behavior	Hypothalamus	[111]
Alzheimer’s disease	Fornix, hypothalamus, and entorhinal cortex	[112]
Chronic pain	Dorsal anterior cingulate cortex, centromedian and parafascicular nuclei, thalamus, periventricular/periaqueductal gray	[113]
Cluster headache	Posterior hypothalamus	[114]
Dementia	Nucleus basalis of Meynert	[115]
Depression	Subcallosal cingulate gyrus, anterior limb of the internal capsule, nucleus accumbens	[116]
Dystonia	Globus pallidus internus, thalamus ventrolateral intermedius nucleus	[117]
Eating disorders	Nucleus accumbens	[118]
Epilepsy	Anterior nucleus of the thalamus; centromedian and parafascicular nuclei; inferior caudate nucleus; subthalamic nucleus	[119]
Huntington’s disease	Globus pallidus externus (in combination with globus pallidus internus)	[120]
Neuropathic deafferentation pain	Ventral posteromedial nucleus/ ventral posterolateral nucleus	[121]
Obesity	Lateral hypothalamic area	[79]
Obsessive-compulsive disorder	Anterior limb of the internal capsule, subthalamic nucleus	[122]
Parkinson’s disease	Globus pallidus internus, subthalamic nucleus	[123]
Tourette syndrome	Nucleus accumbens, globus pallidus internus, centromedian nucleus, ventro-oralis internus	[124]
Tremor	Ventral intermediate thalamus	[125]

**Table 7:** Adverse effects associated with DBS.

Surgical Complications	Long Term Adverse Effects
Hemorrhage (intracerebral/intraventricular)	Dysarthria
Venous infarct	Malignant DBS withdrawal syndrome
Pneumocephalus	Impulse control disorder
Seizures	Paresthesia
Implant infections	Ataxia
Lead dislocation and lead fracture	Diplopia
Post-operative delirium	Twiddler’s syndrome
Worsening dyskinesia	Reduced verbal fluency
Post-operative atelectasis	Changes in memory and executive functions
Pulmonary embolism	Gait disturbances
Deep vein thrombosis	Weight gain

## Data Availability

Not applicable.
